# Impact of COVID-19 on new pharmacotherapy for insomnia: A matched cohort study using the national insurance claims database in Japan

**DOI:** 10.1371/journal.pone.0341416

**Published:** 2026-01-22

**Authors:** Daisuke Miyamori, Shuhei Yoshida, Wataru Omori, Saori Kashima, Masanori Ito

**Affiliations:** 1 Department of General Internal Medicine, Hiroshima University Hospital, Hiroshima, Japan; 2 Department of Psychiatry, NHO Kure Medical Center and Chugoku Cancer Center, Kure, Japan; 3 Center for the Planetary Health and Innovation Science, The IDEC Institute, Hiroshima University, Hiroshima, Japan; 4 Environmental Health Sciences Laboratory, Graduate School of Advanced Science and Engineering, Hiroshima University, Hiroshima, Japan; University of Chicago Division of the Biological Sciences, UNITED STATES OF AMERICA

## Abstract

**Background:**

The COVID-19 pandemic has profoundly affected impacted both physical and mental well-being. This matched cohort study investigated the effects of COVID-19 on pharmacological treatments for insomnia, using Japan’s National Insurance Claims Database.

**Methods:**

Data were matched by age, sex, Charlson Comorbidity Index (CCI) score, and enrollment month. Incidence rate ratios (IRR) and incidence rate differences (IRD) were calculated and compared for insomnia medication initiation, and subgroups based on age and sex categories. Sensitivity analyses were conducted for segmented intervals of 0–4, 5–12, and after 12 months.

**Results:**

The study included approximately 2 million pairs, predominantly women (59.4%), with a median follow-up of 7 months (Interquartile range, 4–12). Initiation of any insomnia medications occurred 77626 and 43142 times in the COVID-19 and control groups, respectively. IRR for new prescriptions was 1.7 times higher in the COVID-19 group (IRR: 1.71, 95% confidence interval [CI] 1.69–1.73), with an IRD of 1,634 events per 1,000,000 person-months (95%CI: 1599–1669). Non-Benzodiazepines and short-acting Benzodiazepines had the highest excess burdens among secondary endpoints. The risk was observed in all age categories, even in under 20 years (younger individuals: IRR: 1.46 95%CI 1.39–1.53, IRD: 380, 95%CI 333–427). Sensitivity analysis confirmed an increased risk over time, even after 12 months (IRD: 752, 95%CI 662–842).

**Conclusion:**

COVID-19 significantly associates with an elevated risk of insomnia medication initiation, emphasizing the necessity for mental health support in post-COVID-19 care. This study offers insights into the pandemic’s influence on pharmacological treatment practices.

## Introduction

The COVID-19 pandemic has significantly impacted global health, causing both acute symptoms and a wide range of post-acute sequelae (PASC). [1–3] Beyond respiratory disease, the pandemic has profoundly influenced sleep patterns and insomnia rates. Several studies have reported increased prevalence of sleep disorders and insomnia during the pandemic [4]. However, trends in pharmacological treatment remains inconsistent. Given this uncertainty, it is crucial to investigate the potential impact of COVID-19 on insomnia treatment needs and hypnotic medication use.

Potential mechanisms for developing post-COVID insomnia include neuroinvasive potential of SARS-CoV-2, autonomic dysfunction, [5]. and, pandemic associated psychological stresses [6], [7]. Post-acute sequelae of SARS-CoV-2 infection (PASC) are persistent symptoms that can last for weeks or even months after initial infection with SARS-CoV-2. [8] These symptoms include fatigue, brain fog, shortness of breath, and other persistent symptoms that can significantly impacting the quality of life and overall health. [9–11] Factors such as male sex, high socioeconomic status, preexisting health conditions, and psychosocial factors may contribute to the development of PASC [12].

Japan presents a unique situation: until recently, cognitive-behavioral therapy for insomnia was seldom reimbursed or recommended, making pharmacotherapy the primary treatment option. [13,14] A cohort study on hospitalized patients showed an increased risk of in Japan; however, studies investigating the risk of initiation of pharmacological treatment for insomnia using population database are scarce. [15] The impact of COVID-19 on mental health, particularly insomnia, represents a potentially huge public health issue. Chronic insomnia is associated with increased risks of depression, anxiety, cardiovascular disease, and all-cause mortality [16,17]. Moreover, the economic burden of insomnia, including healthcare costs and lost productivity, is substantial [18]. Understanding the relationship between COVID-19 and subsequent insomnia medication use can inform public health strategies for post-pandemic mental health support and resource allocation.

This study aimed to evaluate the impact of COVID-19 on the initiation of pharmacological treatments for insomnia. We conducted a matched cohort study using the National Database of Health Insurance Claims and Specific Health Checkups of Japan (NDB). By examining demographic and clinical subgroups, we sought to identify populations at highest risk for developing insomnia following COVID-19 infection, thereby informing targeted interventions and support strategies.

## Methods

### Research Design

This was a matched cohort study using the NDB.

### Data Source

This included outpatient and inpatient information stored at the individual level, which allowed us to track patient-based visits and treatment. The claims database included information on age, sex, diagnoses based on the International Statistics Classification of Diseases and Related Health Problems, Tenth Revision codes (ICD-10) with Japanese texts, and drugs dispensed based on the Anatomical Therapeutic Chemical (ATC) Classification System. The data for this research were accessed on 16/08/2024.

### Study Participants

Participants were individuals from five prefectures in Japan (Kyoto, Osaka, Hyogo, Okayama, and Hiroshima), who used the National Insurance Claims Database between January 2020 and December 2022. The area covered 20.5 million people and approximately 16% of Japan’s population. In the five prefectures included in this study, the age-demographic composition was similar to that of the nation as a whole, and the cost frequency for psychiatric disorders as of 2021 did not show extreme bias [19] (S1 and S2 Tables).

### Outcome

The outcome of interest of this study was the initiation of prescription for insomnia which are available for outpatients in Japan, with insomnia being the main indication, and other indications such as anxiety and epilepsy being excluded. We categorize the drug as follows: Melatonin Receptor Agonist (MRA), Non-Benzodiazepine Hypnotic (non-BZO), benzodiazepine (short-acting [SA-BZO], intermediate-/long-acting [ILA-BZO]), Orexin Receptor Antagonist (ORA) and Barbiturates. The composite endpoint included the initiation of medication in these categories. The secondary endpoint was the new prescription for each drug category. A detailed list of the medications included in each category is provided in S3 Table.

### Exposure

Subjects diagnosed with COVID-19 during the study period were assigned to the COVID-19 group. The government exempted individuals diagnosed with COVID-19 during the observation period from incurring medical expenses. In this study, the public expense number on payment was used to determine the exposure status [20].

### Inclusion and exclusion criteria

All individuals who were insured and had access to healthcare from January 2020, when COVID-19 was prevalent, to December 2022, were included in this study. We excluded those who did not have a 1-year look-back period before the index month. We also excluded those who had received hypnotics drugs before the index month during the 1-year pre-assessment period. Details of the data assessment in this study are provided in S1 Fig.

### Data collection

The NDB served as the primary data source, providing details such as participant’s age, sex, and ICD-10 codes for registered diseases and information on their prescription history, including specific drug names. Japan’s universal health insurance system features a detailed NDB that thoroughly records each outpatient appointment, hospital stay, diagnostic code, and medication prescribed for all residents. The Charlson Comorbidity Index (CCI) was determined utilizing the ICD-10 codes derived from the registered disease codes prior to the index month for both the infected and control groups. [21,22]

### Data Matching

The data organization was based on four variables: age category, sex, CCI total score, and month of enrollment in the insurance claims database. The month the participants were infected with COVID-19 was designated the index month. To ensure an accurate matching algorithm, we sought controls from the claims database that perfectly matched the cases for all four variables. For each month, controls matched on all four criteria were identified and paired randomly with cases. Once matched, participants were used exclusively once and were not reused in subsequent matching. The validity of the matching was assessed by comparing comorbidities between groups using standardized mean differences (SMD), with an SMD ≤ 0.1 considered indicative of balanced groups.

### Follow-up of the cohorts

The maximum follow-up period in this study was 24 months. All individuals in the matched cohort were tracked from the index date to the initiation of new pharmacotherapy for insomnia or the conclusion of the follow-up period, whichever occurred first.

### Statistical analyses

We used the Kaplan–Meier estimator to calculate the cumulative incidence of the composite endpoint that occurred over time, starting from the index date. Subsequently, we assessed the incidence rate difference (IRD) and incidence rate ratio (IRR) per 1,000,000 person-months between the COVID-19 and control groups, along with 95% confidence intervals (CI). For subgroup analysis, we calculated the IRD and IRR of the composite outcomes between the COVID-19 and Control groups for subgroups of age and sex categories. In addition, a sensitivity analysis was conducted to assess the robustness of the main and subgroup analyses by splitting the study period into three intervals: the first 4 months, between 5 and 12 months, and after 12 months since the COVID-19 infection or index month.

### Ethical consideration

The study protocol was reviewed and approved by the Epidemiological Research Committee of Hiroshima University (approval number: E2022-0024-01). The data for this research were owned by Ministry of Health, Labor, and Welfare in Japan (MHLW) and obtained by the authors with ethical approval (approval number: 1502).

## Results

**Fig 1:**
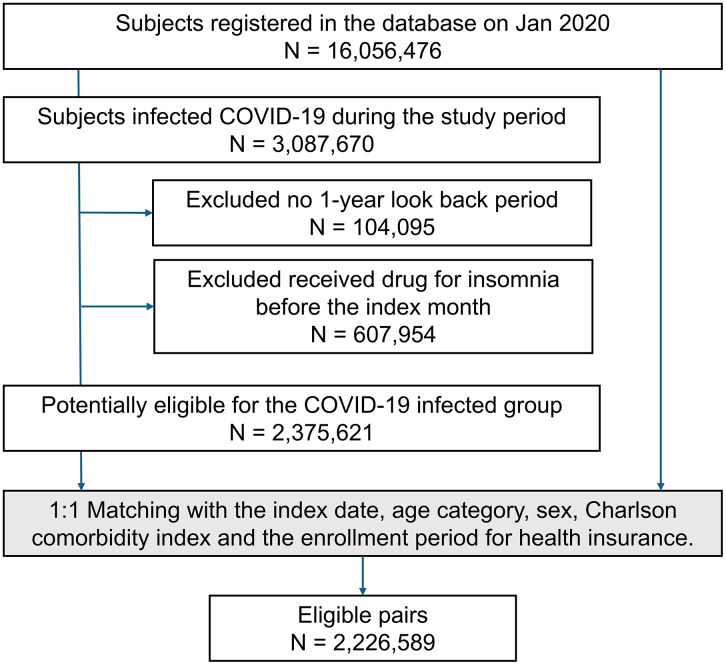
Flow chart of Study Participants. Among roughly 16 million out of an estimated 3 million individuals, approximately 16 million were infected. After excluding 607,954 individuals who had received hypnotics medication within the past year, 2,375,621 subjects were eligible for the COVID-19 group and the matched cohort study. Lastly, 2,226,589 pairs were matched and analyzed. In the matching process, age categories were divided into groups every 1 year for subjects under 10 years old, and in increments of 5 years for subjects aged 10 years and older. The Charlson Comorbidity Index was matched using total scores.

Fig 1 shows the flowchart of this study. This study included approximately 16 million participants, with 3 million individuals infected with COVID-19. After excluding individuals who did not have a 1-year look-back period and individuals who had already received prescriptions for insomnia, A COVID-19 group of 2375621 individuals was matched with a non-infected group. After matching for age category, gender, CCI, and insurance enrollment month, a total of 2,226,589 pairs were included in the study.

Table 1 presents the baseline characteristics of 4,453,178 study participants, equally divided between the control group and the COVID-19 group (2,226,589 each). The table shows demographic information and comorbidities for both groups. The standardized mean difference (SMD) is used to assess the balance between groups, with values close to zero indicating good balance. The participants were predominantly women (54.94%), and the largest age category was the 70s.

**Table 1:** Baseline characteristics of study participants.

	Total	Control group	COVID-19 group	SMD
	N = 4,453,178	N = 2,226,589	N = 2,226,589	
Women (%)	2,443,966 (54.9%)	1,221,983 (54.9%)	1,221,983 (54.9%)	0
Age category (%)				0
0–4 years	315,238 (7%)	157,619 (7%)	157,619 (7%)	
5–9 years	259,698 (6%)	129,849 (6%)	129,849 (6%)	
10–14 years	186,020 (4%)	93,010 (4%)	93,010 (4%)	
15–19 years	209,464 (5%)	104,732 (5%)	104,732 (5%)	
20–24 years	190,654 (4%)	95,327 (4%)	95,327 (4%)	
25–29 years	203,532 (5%)	101,766 (5%)	101,766 (5%)	
30–34 years	216,556 (5%)	108,278 (5%)	108,278 (5%)	
35–39 years	217,038 (5%)	108,519 (5%)	108,519 (5%)	
40–44 years	205,936 (5%)	102,968 (5%)	102,968 (5%)	
45–49 years	258,474 (6%)	129,237 (6%)	129,237 (6%)	
50–54 years	266,552 (6%)	133,276 (6%)	133,276 (6%)	
55–59 years	231,032 (5%)	115,516 (5%)	115,516 (5%)	
60–64 years	229,702 (5%)	114,851 (5%)	114,851 (5%)	
65–69 years	239,540 (5%)	119,770 (5%)	119,770 (5%)	
70–74 years	433,070 (10%)	216,535 (10%)	216,535 (10%)	
75–79 years	285,654 (6%)	142,827 (6%)	142,827 (6%)	
80–84 years	277,520 (6%)	138,760 (6%)	138,760 (6%)	
85 + years	227,498 (5%)	113,749 (5%)	113,749 (5%)	
CCI (%)				0
0	2,316,576 (52%)	1,158,288 (52%)	1,158,288 (52%)	
1	879,556 (20%)	439,778 (20%)	439,778 (20%)	
2–3	740,272 (17%)	370,136 (17%)	370,136 (17%)	
4 or above	516,774 (12%)	258,387 (12%)	258,387 (12%)	
Comorbidities				
AMI	66,427 (1.5%)	33,129 (1.5%)	33,298 (1.5%)	0.001
CHF	462,268 (10.4%)	223,463 (10.0%)	238,805 (10.7%)	0.02
PVD	410,040 (9.2%)	203,022 (9.1%)	207,018 (9.3%)	0.01
CEVD	493,194 (11.1%)	242,035 (10.9%)	251,159 (11.3%)	0.01
Dementia	90,640 (2.0%)	36,047 (1.6%)	54,593 (2.5%)	0.06
CPD	339,288 (7.6%)	161,171 (7.2%)	178,117 (8.0%)	0.03
Rheumatoid	139,994 (3.1%)	68,156 (3.1%)	71,838 (3.2%)	0.01
PUD	849,366 (19.1%)	413,469 (18.6%)	435,897 (19.6%)	0.03
Diabetes	271,083 (6.1%)	138,150 (6.2%)	132,933 (6.0%)	−0.01
Liver disease	857,180 (19.2%)	430,785 (19.3%)	426,395 (19.2%)	−0.01
Cancer	361,892 (8.1%)	185,429 (8.3%)	176,463 (7.9%)	−0.01
HP/PAPL	38,807 (0.9%)	18,656 (0.8%)	20,151 (0.9%)	0.01
RD	134,674 (3.0%)	63,942 (2.9%)	70,732 (3.2%)	0.02
AIDS	1,970 (0.0%)	1,078 (0.0%)	892 (0.0%)	−0.004

CCI: Charlson comorbidity index. AMI, acute myocardial infarction; CHF, congestive heart failure; PVD, peripheral vascular disease; CEVD, cerebrovascular disease; Dementia, dementia; CPD, chronic pulmonary disease; Rheumatoid, rheumatoid and other connective tissue diseases (including collagen vascular diseases); PUD, peptic ulcer disease; Diabetes, diabetes mellitus; Liver disease, chronic liver disease; Cancer, malignant neoplasm; HP/PAPL, hemiplegia/paraplegia; RD, renal disease; AIDS, acquired immune deficiency syndrome.

**Fig 2:**
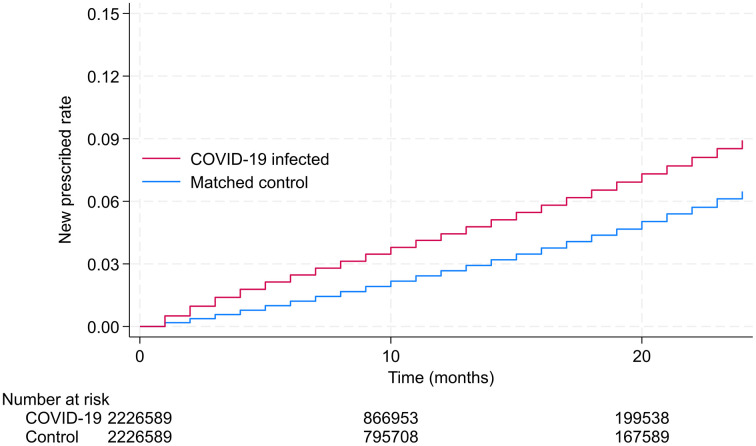
Kaplan-Meier Analysis of New Prescription Rates for Insomnia. Cumulative incidence of new pharmacotherapy for insomnia among individuals with COVID-19 (blue line) and a matched non-infected control group (red line). The p-value for the log-rank test was < 0.001, indicating a statistically significant difference between the two groups. The shaded areas around the lines represent 95% confidence intervals.

Fig 2 shows the Kaplan–Meier curves for participants infected with COVID-19 and matched controls for all participants, and Table 2 shows the IRR and IRD for the composite endpoint and secondary endpoint. The cumulative incidence of pharmacotherapy for insomnia continuously increased during the study period. The included 2.2 million pairs were followed for a median duration of 7 months (Interquartile range 4–12). Of the 2,226,589 individuals in each group, 77,626 and 43,142 cases of the composite endpoint occurred in the COVID-19 and control groups, respectively, over a 24-month observational period. The IRR was 1.71 (95% CI, 1.69–1.73) and IRD was 1,634 events per 1,000,000 person-months (95% CI, 1599–1669). The analyses of secondary endpoints showed an increased risk of prescription for any of the hypnotic categories in the COVID-19 group compared with the control group. During the study period, ORA and non-BZO were the most frequently prescribed, and the IRD was 791 (95% CI: 767–815) and 614 (95% CI: 592–636) events per 1,000,000 person-months, and the IRR was 1.73 (95% CI: 1.70–1.76) and 1.70 (95% CI: 1.67–1.82), respectively. In the subgroup analyses, the risk of new prescriptions significantly increased across categories.

**Table 2:** Cumulative Incidences of Pharmacotherapy for Insomnia after the COVID-19 infection in the Matched Cohort Design for Composite endpoint, secondary endpoint and subgroup categories.

	No. of pairs	No. of Events in COVID-19 Group	No. of Events in Control Group	Cumulative Incidence (No. of Events per 1 000 000 Person months)	
				*Ratio (95% CI)*	*Difference (95% CI)*
Composite endpoint	2226589	77626	43142	1.71 (1.69–1.73)	1634 (1599–1669)
Secondary endpoints					
SA-BZO	2226589	15945	8560	1.75 (1.71–1.80)	341 (326–357)
ILA-BZO	2226589	4263	2309	1.73 (1.65–1.82)	90 (81–98)
Non-BZO	2226589	29860	16585	1.70 (1.67–1.73)	614 (592–636)
MRA	2226589	12022	5708	1.98 (1.92–2.04)	296 (283–310)
ORA	2226589	37341	20324	1.73 (1.70–1.76)	791 (767–815)
Barbiturates	2226589	48	15	3.00 (1.65 – 5.77)	1.59 (0.80 to 2.37)

No.: number; CI: confidence interval; MRA: melatonin receptor agonist; non-BZO: Non-Benzodiazepine Hypnotics; SA-BZO: short-acting benzodiazepines; ILA-BZO: Intermediate/long-acting benzodiazepines; ORA: orexin receptor agonist.

**Fig 3:**
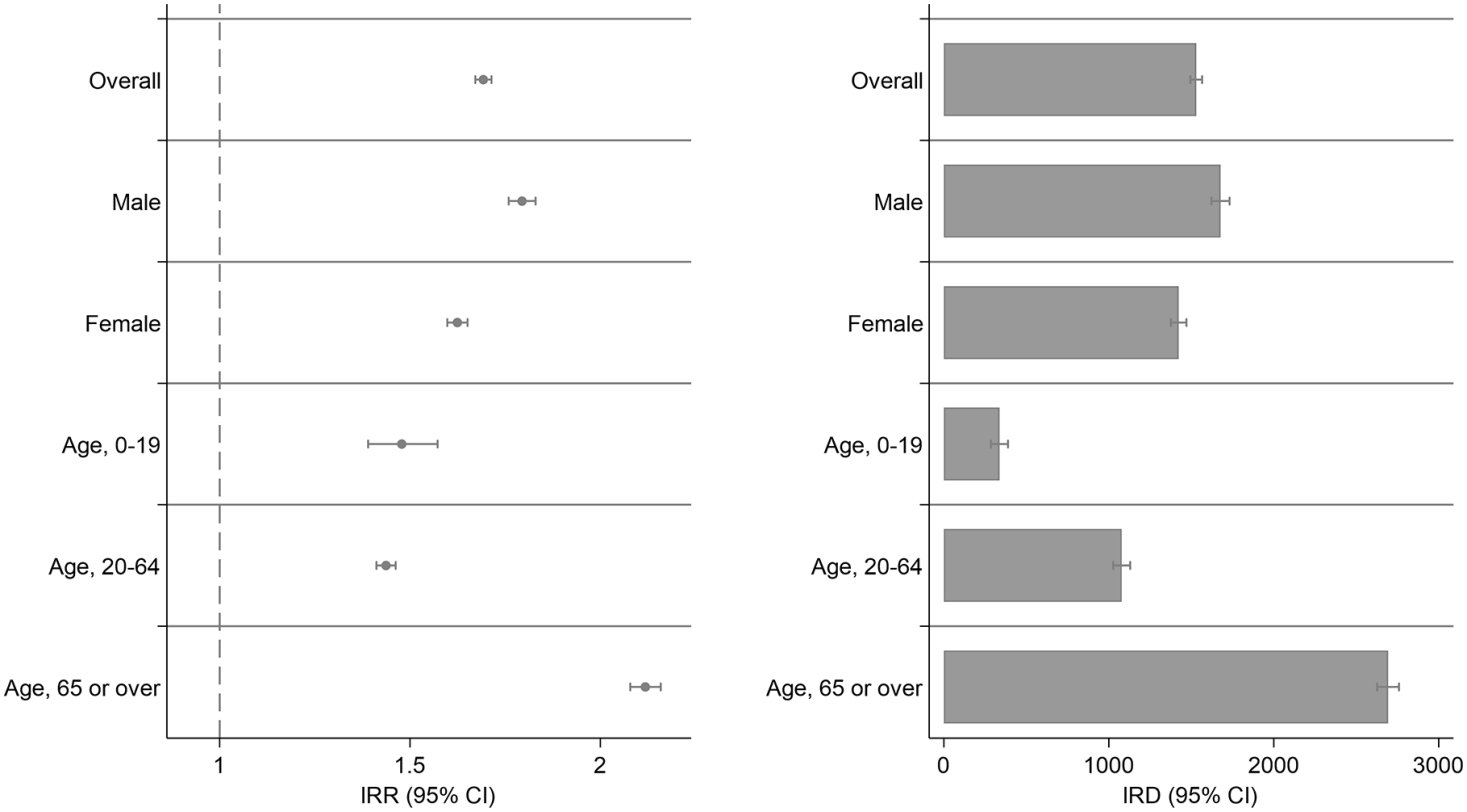
Subgroup analysis for incidence rate difference and ratio. This figure presents the Incidence Rate Ratios (IRR) with 95% Confidence Intervals (CI) on the left panel and the Incidence Rate Differences (IRD) with 95% Confidence Intervals (CI) on the right panel, stratified by demographic and clinical subgroups. Left Panel (IRR, 95% CI)**:** The IRR compares the incidence rates between the exposure group and the control group within each subgroup. An IRR greater than 1 indicates a higher incidence rate in the exposure group compared to the control group. Right Panel (IRD, 95% CI): The IRD shows the absolute difference in incidence rates per 1,000,000 individuals between the exposure and control groups within each subgroup. A positive IRD indicates a higher incidence in the exposure group. The subgroups are the same as in the left panel.

Fig 3 shows the IRR and IRD for the subgroups; the numbers are shown in S4 Table. The IRR and IRD were elevated in all subgroups, consistent with the main analysis.

**Fig 4:**
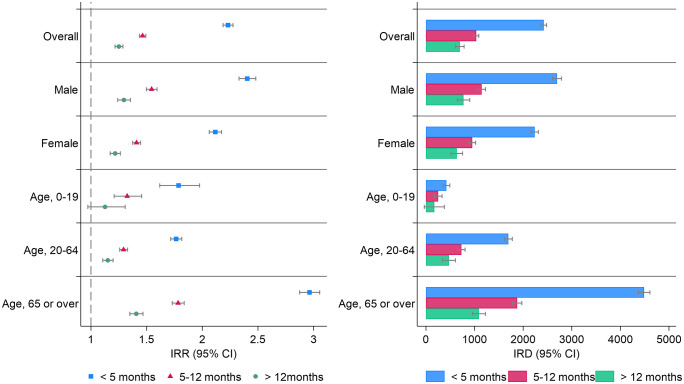
Sensitivity analysis for incidence rate difference and ratio across segmented intervals. This graph illustrates the sensitivity analysis of the incidence rate difference (IRD) and incidence rate ratio (IRR) across various categories based on age, sex, and the Charlson Comorbidity Index. The analysis is divided into four distinct time intervals: 0-4 months (Blue), 5–12 months (Red), and more than 12 months (Green). Left panel (IRR): The markers in the left panel represent the IRR across the time intervals, illustrating the relative risk of the incidence rate between the exposed and unexposed groups within each category. Right panel (IRD): The height of the bars in the right panel indicates the IRD, representing the absolute difference in incidence rates between the groups within each time interval. The vertical lines attached to the bars and plots represent the 95% confidence intervals for IRD and IRR. IRR, incidence rate ratio; IRD, incidence rate difference; CCI, Charlson comorbidity index; CI, confidence interval.

The sensitivity analysis presented in Fig 4 shows the incidence risk ratios and differences for the composite endpoint across the overall and subgroup categories, into three distinct time intervals: within 4 months, between 5 and 12 months, and after 1 year; the numbers are shown in S5 Table. The incidence risk ratios within 4 months, between 5 and 12 months, and after 12 months were 2.33 (95% CI: 2.28–2.37), 1.46 (95% CI: 1.43–1.48) and 1.25 (95% CI: 1.21–1.28), respectively. The incidence rate of new prescriptions for insomnia was higher in the COVID-19 group than in the control group for all subgroup categories, even after the 1-year follow-up period. Sensitivity analyses indicated that the frequency exhibited a declining trend over time following infection, yet remained significantly higher than that in the control group.

## Discussion

This study aimed to investigate the incidence of initiating pharmacological treatments for insomnia after COVID-19 and compare it with that in individuals without infections. Our findings indicate that the incidence of initiating insomnia medications increased approximately 1.7 times in individuals with post-COVID-19 infection compared with those without infections. Subgroup analyses showed that this increased incidence was consistently observed across all age groups, including children and adolescents, and in both women and men (Fig 3 and S4 Table). The increased incidence of new insomnia medications persisted throughout the study period, even after 1-year of infection.

Studies reported a sharp rise in hypnotics after COVID-19, with an increase of 0.23 boxes per 1000 persons, [4] and higher insomnia diagnosis risk of 1.92 times at six months and 2.46 times at one year. [23] Additionally, 30% of severe PASC patients needed sleep-inducing drugs long-term. [24] Using the Population database, this study found the risk after COVID-19 persisted beyond 2 years. In Japan, cognitive-behavioral therapy for insomnia (CBT-I) is not covered by insurance, and pharmacotherapy remains the predominant treatment. [13] Therefore, the prescription of hypnotics in this study may reflects the onset of new cases of insomnia.

Insomnia exacerbates physical and psychiatric disorders, often with anxiety, depression, and increased stress. Chronic insomnia is linked to cognitive decline, reduced quality of life, and higher risks of injuries, suicidal thoughts, cardiovascular issues, diabetes, and cancer. [25–28] In addition, the mechanism of insomnia in PASC involves multiple biological and psychological factors. [21,29,30] Insomnia in PASC patients likely results from neuroinflammatory processes, autonomic dysfunction, direct viral effects on the central nervous system, psychological stressors, circadian rhythm disruptions, and physical symptoms. [31] Furthermore, the substantial increase in prescriptions of orexin receptor antagonists and non-benzodiazepine hypnotics observed in this study may have important implications for healthcare resource use, underscoring the need to expand access to cost-effective non-pharmacological interventions in Japan. The COVID-19 pandemic heightened challenges, impacting all generations and increasing healthcare burdens and economic losses due to reduced productivity. [32]

### Strengths and limitations

A key strength of this study is that it used a national insurance claims database, including 2.2 million COVID-19 and control individuals. Therefore, a comprehensive evaluation of personal-level risk factors for insomnia medication can be accomplished by utilizing a substantial, well-matched cohort database, which minimizes bias and presents more precise outcomes. Second, the certainty of the confirmation of COVID-19 infection would be accurate. Because the reimbursement for COVID-19 infection is paid by public funds, all insurance codes for the diagnosis and treatment of patients diagnosed with COVID-19 were recorded. Moreover, during the study period, all COVID-19 infections were mandatorily reported in Japan.

This study has limitations. First, our study lacked data on COVID-19 severity, which may have influenced outcomes. Second, selection bias may exist due to misclassification of COVID-19 infection and outcome status. Individuals with mild COVID-19 symptoms who were not diagnosed might have been classified as controls, although this bias might work as a point estimate into null. Insomnia may be more frequently prescribed in patients with post-COVID-19 conditions, potentially amplifying results. Third, we did not account for vaccination, economic, and employment statuses. Fourth, the codes identified have not undergone validation. Research assessing Japanese diagnostic codes for hospitalized patients showed sensitivity and specificity of 78.9% and 93.2%, respectively. [33]. Fifth, despite matching on key variables, the study may be subject to residual confounding from unmeasured factors, such as health seeking behaviors. A potential limitation of this study is the increased likelihood of healthcare-seeking behavior among individuals with COVID-19 compared to those without the disease. [34,35] The emphasis on early detection and isolation in public health messaging may have led to higher rates of medical attention-seeking. [35] These factors could have influenced the observed patterns in healthcare utilization and should be considered when interpreting the results. Sixth, this study sampled patients in only five prefectures, not all of Japan, and COVID-affected and insomnia patient rates may differ across prefectures. Similarly, generalizability is limited in countries with different cultural backgrounds and treatment policies. Finally, the possibility that medications were prescribed for reasons other than insomnia cannot be excluded entirely. [36,37] Future research should consider multi-country cohort studies to explore the generalizability of our findings across different healthcare systems and cultural contexts. Longitudinal research with extended follow-up periods is necessary to determine the persistence of insomnia symptoms in patients post-COVID-19 and assess the efficacy and safety of pharmacotherapeutic interventions.

## Conclusions

In conclusion, our findings demonstrated a significant association between COVID-19 and a heightened risk of initiating insomnia pharmacotherapy. This underscores the urgent need to integrate mental health support into the post-COVID-19 care protocols. Ensuring timely intervention is crucial in managing mental health issues in the context of COVID-19. Our study contributes to a more detailed and contextual understanding of the impact of COVID-19 on pharmacological treatment practices.

Table of contents: This study on the impact of COVID-19 on new insomnia pharmacotherapy in Japan, using a matched cohort from the National Insurance Claims Database, revealed a significant 1.7-fold increase in insomnia medication initiation among COVID-19 patients compared with that in controls. Older individuals and those with higher Charlson Comorbidity Index scores are at high risk; the elevated risk persists beyond one year post-infection. This underscores the pandemic’s impact on mental health and the need for integrated support in post-COVID-19 care.

## Supporting information

S1 Table Population of 5 Prefectures and Japan by Age Group and Sex. (DOCX)

S2 Table Average per capita medical expenses related to mental illness in targeted prefectures. (DOCX)

S3 Table ATC code, categories and drug names included in this study. (DOCX)

S4 Table Incidence Rate Ratios and Differences of Pharmacotherapy for Insomnia after the COVID-19 infection in the Matched Cohort Design for Composite endpoint and subgroup categories. (DOCX)

S5 Table Sensitivity Analysis of Pharmacotherapy for Insomnia after the COVID-19 infection in the Matched Cohort Design for Composite endpoint and subgroup categories. (DOCX)

S1 Fig Illustration of this study design. (DOCX)

## References

[1] Estrela M, Silva TM, Gomes ER, Piñeiro M, Figueiras A, Roque F, et al. Prescription of anxiolytics, sedatives, hypnotics and antidepressants in outpatient, universal care during the COVID-19 pandemic in Portugal: A nationwide, interrupted time-series approach. J Epidemiol Community Health. 2022;76(4):335–40. doi: 10.1136/jech-2021-216732 34625519

[2] Renaud-Charest O, Lui LMW, Eskander S, Ceban F, Ho R, Di Vincenzo JD, et al. Onset and frequency of depression in post-COVID-19 syndrome: A systematic review. J Psychiatr Res. 2021;144:129–37. doi: 10.1016/j.jpsychires.2021.09.054 34619491 PMC8482840

[3] Bueno-Notivol J, Gracia-García P, Olaya B, Lasheras I, López-Antón R, Santabárbara J. Prevalence of depression during the COVID-19 outbreak: A meta-analysis of community-based studies. Int J Clin Health Psychol. 2021;21(1):100196. doi: 10.1016/j.ijchp.2020.07.007 32904715 PMC7458054

[4] Tiger M, Castelpietra G, Wesselhoeft R, Lundberg J, Reutfors J. Utilization of antidepressants, anxiolytics, and hypnotics during the COVID-19 pandemic. Transl Psychiatry. 2024;14(1):175. doi: 10.1038/s41398-024-02894-z 38575574 PMC10995182

[5] Song E, Zhang C, Israelow B, Lu-Culligan A, Prado AV, Skriabine S, et al. Neuroinvasion of SARS-CoV-2 in human and mouse brain. J Exp Med. 2021;218(3):e20202135. doi: 10.1084/jem.20202135 33433624 PMC7808299

[6] Teague T, Debian A, Kokonda M, Malhotra S, Arentson-Lantz E, Shaib F, et al. 0681 Association of poor sleep with stress, anxiety, emotional support, social isolation, and depression during the COVID-19 pandemic. Sleep. 2022;45(Supplement_1):A298–9. doi: 10.1093/sleep/zsac079.677

[7] Rochmawati E, Iskandar AC, Kamilah F. Persistent symptoms among post-COVID-19 survivors: A systematic review and meta-analysis. J Clin Nurs. 2022. doi: 10.1111/jocn.16471 36426658

[8] Soriano JB, Murthy S, Marshall JC, Relan P, Diaz JV. WHO Clinical Case Definition Working Group on Post-COVID-19 Condition. A clinical case definition of post-COVID-19 condition by a Delphi consensus. Lancet Infect Dis. 2022;22(4):e102–7. doi: 10.1016/S1473-3099(21)00703-9 34951953 PMC8691845

[9] Whitaker M, Elliott J, Chadeau-Hyam M, Riley S, Darzi A, Cooke G, et al. Persistent COVID-19 symptoms in a community study of 606,434 people in England. Nat Commun. 2022;13(1):1957. doi: 10.1038/s41467-022-29521-z 35413949 PMC9005552

[10] Kim Y, Bitna Ha, Kim S-W, Chang H-H, Kwon KT, Bae S, et al. Post-acute COVID-19 syndrome in patients after 12 months from COVID-19 infection in Korea. BMC Infect Dis. 2022;22(1):93. doi: 10.1186/s12879-022-07062-6 35086489 PMC8793328

[11] Mazza MG, Palladini M, De Lorenzo R, Bravi B, Poletti S, Furlan R, et al. One-year mental health outcomes in a cohort of COVID-19 survivors. J Psychiatr Res. 2022;145:118–24. doi: 10.1016/j.jpsychires.2021.11.031 34894521 PMC8607816

[12] Zeng N, Zhao Y-M, Yan W, Li C, Lu Q-D, Liu L, et al. A systematic review and meta-analysis of long term physical and mental sequelae of COVID-19 pandemic: Call for research priority and action. Mol Psychiatry. 2023;28(1):423–33. doi: 10.1038/s41380-022-01614-7 35668159 PMC9168643

[13] Takaesu Y, Sakurai H, Aoki Y, Takeshima M, Ie K, Matsui K, et al. Treatment strategy for insomnia disorder: Japanese expert consensus. Front Psychiatry. 2023;14:1168100. doi: 10.3389/fpsyt.2023.1168100 37229388 PMC10203548

[14] Hayashi Y, Yoshinaga N, Sasaki Y, Tanoue H, Yoshimura K, Kadowaki Y, et al. How was cognitive behavioural therapy for mood disorder implemented in Japan? A retrospective observational study using the nationwide claims database from FY2010 to FY2015. BMJ Open. 2020;10(5):e033365. doi: 10.1136/bmjopen-2019-033365 32376747 PMC7223011

[15] Murata F, Maeda M, Ishiguro C, Fukuda H. Acute and delayed psychiatric sequelae among patients hospitalised with COVID-19: A cohort study using LIFE study data. Gen Psychiatr. 2022;35(3):e100802. doi: 10.1136/gpsych-2022-100802 35846486 PMC9214348

[16] Khan MS, Aouad R. The effects of insomnia and sleep loss on cardiovascular disease. Sleep Med Clin. 2022;17(2):193–203. doi: 10.1016/j.jsmc.2022.02.008 35659073

[17] Ge L, Guyatt G, Tian J, Pan B, Chang Y, Chen Y, et al. Insomnia and risk of mortality from all-cause, cardiovascular disease, and cancer: Systematic review and meta-analysis of prospective cohort studies. Sleep Med Rev. 2019;48:101215. doi: 10.1016/j.smrv.2019.101215 31630016

[18] The economic burden of insomnia: Direct and indirect costs for individuals with insomnia syndrome, insomnia symptoms, and good sleepers. Sleep. 2009. doi: 10.5665/sleep/32.1.55 PMC2625324 19189779

[19] Minisitry of Health Labour and Welfare. Database on regional disparities in medical expenditure (Iryohi map). 2023. https://www.mhlw.go.jp/content/iryohi_r04_kiso.xlsx

[20] Ministry of Health Labour and Welfare. Response to COVID-19 after the classification change. 2023. https://www.mhlw.go.jp/stf/seisakunitsuite/bunya/0000164708_00079.html

[21] Charlson ME, Pompei P, Ales KL, MacKenzie CR. A new method of classifying prognostic comorbidity in longitudinal studies: Development and validation. J Chronic Dis. 1987;40(5):373–83. doi: 10.1016/0021-9681(87)90171-8 3558716

[22] Quan H, Sundararajan V, Halfon P, Fong A, Burnand B, Luthi J-C, et al. Coding algorithms for defining comorbidities in ICD-9-CM and ICD-10 administrative data. Med Care. 2005;43(11):1130–9. doi: 10.1097/01.mlr.0000182534.19832.83 16224307

[23] Taquet M, Geddes JR, Husain M, Luciano S, Harrison PJ. 6-month neurological and psychiatric outcomes in 236 379 survivors of COVID-19: A retrospective cohort study using electronic health records. Lancet Psychiatry. 2021;8(5):416–27. doi: 10.1016/S2215-0366(21)00084-5 33836148 PMC8023694

[24] Batool-Anwar S, Fashanu OS, Quan SF. Long-term Effects of COVID-19 on Sleep Patterns. Thorac Res Pract. 2025;26(1):9–16. doi: 10.5152/ThoracResPract.2024.24013 39663632 PMC11784924

[25] Liu RT, Steele SJ, Hamilton JL, Do QBP, Furbish K, Burke TA, et al. Sleep and suicide: A systematic review and meta-analysis of longitudinal studies. Clin Psychol Rev. 2020;81:101895. doi: 10.1016/j.cpr.2020.101895 32801085 PMC7731893

[26] Chalet F-X, Saskin P, Ahuja A, Thompson J, Olopoenia A, Modi K, et al. The Associations between Insomnia Severity and Health Outcomes in the United States. J Clin Med. 2023;12(6):2438. doi: 10.3390/jcm12062438 36983438 PMC10053531

[27] Garrigues E, Janvier P, Kherabi Y, Le Bot A, Hamon A, Gouze H, et al. Post-discharge persistent symptoms and health-related quality of life after hospitalization for COVID-19. J Infect. 2020;81(6):e4–6. doi: 10.1016/j.jinf.2020.08.029 32853602 PMC7445491

[28] Whiteside DM, Basso MR, Naini SM, Porter J, Holker E, Waldron EJ, et al. Outcomes in post-acute sequelae of COVID-19 (PASC) at 6 months post-infection Part 1: Cognitive functioning. Clin Neuropsychol. 2022;36(4):806–28. doi: 10.1080/13854046.2022.2030412 35130818

[29] Mohandas S, Jagannathan P, Henrich TJ, Sherif ZA, Bime C, Quinlan E, et al. Immune mechanisms underlying COVID-19 pathology and post-acute sequelae of SARS-CoV-2 infection (PASC). Elife. 2023;12:e86014. doi: 10.7554/eLife.86014 37233729 PMC10219649

[30] Brown LA, Ballentine E, Zhu Y, McGinley EL, Pezzin L, Abramoff B. The unique contribution of depression to cognitive impairment in Post-Acute Sequelae of SARS-CoV-2 infection. Brain Behav Immun Health. 2022;22:100460. doi: 10.1016/j.bbih.2022.100460 35403066 PMC8983478

[31] Lee M-H, Perl DP, Nair G, Li W, Maric D, Murray H, et al. Microvascular Injury in the Brains of Patients with Covid-19. N Engl J Med. 2021;384(5):481–3. doi: 10.1056/NEJMc2033369 33378608 PMC7787217

[32] Morikawa M. Productivity of working from home during the COVID-19 pandemic: Evidence from an employee survey. Covid Economics. 2020;49:123–39.

[33] Yamana H, Moriwaki M, Horiguchi H, Kodan M, Fushimi K, Yasunaga H. Validity of diagnoses, procedures, and laboratory data in Japanese administrative data. J Epidemiol. 2017;27(10):476–82. doi: 10.1016/j.je.2016.09.009 28142051 PMC5602797

[34] Hua J, Chen R, Zhao L, Wu X, Guo Q, He C, et al. Epidemiological features and medical care-seeking process of patients with COVID-19 in Wuhan, China. ERJ Open Res. 2020;6(2):00142–2020. doi: 10.1183/23120541.00142-2020 32363205 PMC7184112

[35] Zanin M, Xiao C, Liang T, Ling S, Zhao F, Huang Z, et al. The public health response to the COVID-19 outbreak in mainland China: A narrative review. J Thorac Dis. 2020;12(8):4434–49. doi: 10.21037/jtd-20-2363 32944357 PMC7475588

[36] Vehar S, Boushra M, Ntiamoah P, Biehl M. Post-acute sequelae of SARS-CoV-2 infection: Caring for the “long-haulers”. Cleve Clin J Med. 2021;88(5):267–72. doi: 10.3949/ccjm.88a.21010 33941600

[37] Quan SF, Weaver MD, Czeisler MÉ, Barger LK, Booker LA, Howard ME, et al. Association of obstructive sleep apnea with post-acute sequelae of SARS-CoV-2 infection. Am J Med. 2024;137(6):529-537.e3. doi: 10.1016/j.amjmed.2024.02.023 38401674 PMC11144080

