# Supplementary material for: Impact of COVID-19 on new pharmacotherapy for insomnia: A matched cohort study using the national insurance claims database in Japan
Source: PLoS One. 2026 Jan 22;21(1):e0341416. doi: 10.1371/journal.pone.0341416 (PMC12826487; doi:10.1371/journal.pone.0341416)
Supplement: S1 Table — (DOCX) [file pone.0341416.s001.docx]

Supplementary Table 1. Populations in this study and Japan by Age Group and Sex

| **Age Group (years)** | **Population in Japan (N=126,146,000), n (%)** | **Study Population (N=21,569,000), n (%)** | **p-value** | **Men: Japan (N=61,350,000), n (%)** | **Men: Study Population (N=10,332,000), n (%)** | **p-value** | **Women: Japan (N=64,797,000), n (%)** | **Women: Study Population (N=11,237,000), n (%)** | **p-value** |
| --- | --- | --- | --- | --- | --- | --- | --- | --- | --- |
| 0–4 | 4,541 (3.6%) | 782 (3.6%) | 0.19 | 2,325 (3.8%) | 400 (3.9%) | 0.83 | 2,217 (3.4%) | 381 (3.4%) | 0.19 |
| 5–9 | 5,114 (4.1%) | 875 (4.1%) |  | 2,620 (4.3%) | 447 (4.3%) |  | 2,494 (3.8%) | 429 (3.8%) |  |
| 10–14 | 5,376 (4.3%) | 924 (4.3%) |  | 2,756 (4.5%) | 474 (4.6%) |  | 2,620 (4.0%) | 450 (4.0%) |  |
| 15–19 | 5,706 (4.5%) | 1,006 (4.7%) |  | 2,928 (4.8%) | 513 (5.0%) |  | 2,779 (4.3%) | 493 (4.4%) |  |
| 20–24 | 6,320 (5.0%) | 1,145 (5.3%) |  | 3,234 (5.3%) | 574 (5.6%) |  | 3,086 (4.8%) | 571 (5.1%) |  |
| 25–29 | 6,384 (5.1%) | 1,093 (5.1%) |  | 3,279 (5.3%) | 547 (5.3%) |  | 3,105 (4.8%) | 546 (4.9%) |  |
| 30–34 | 6,714 (5.3%) | 1,124 (5.2%) |  | 3,431 (5.6%) | 564 (5.5%) |  | 3,283 (5.1%) | 561 (5.0%) |  |
| 35–39 | 7,498 (5.9%) | 1,232 (5.7%) |  | 3,806 (6.2%) | 611 (5.9%) |  | 3,692 (5.7%) | 621 (5.5%) |  |
| 40–44 | 8,476 (6.7%) | 1,411 (6.5%) |  | 4,299 (7.0%) | 697 (6.7%) |  | 4,178 (6.4%) | 714 (6.4%) |  |
| 45–49 | 9,868 (7.8%) | 1,728 (8.0%) |  | 4,994 (8.1%) | 854 (8.3%) |  | 4,875 (7.5%) | 872 (7.8%) |  |
| 50–54 | 8,738 (6.9%) | 1,516 (7.0%) |  | 4,394 (7.2%) | 747 (7.2%) |  | 4,344 (6.7%) | 770 (6.9%) |  |
| 55–59 | 7,940 (6.3%) | 1,336 (6.2%) |  | 3,967 (6.5%) | 655 (6.3%) |  | 3,973 (6.1%) | 683 (6.1%) |  |
| 60–64 | 7,442 (5.9%) | 1,200 (5.6%) |  | 3,677 (6.0%) | 584 (5.7%) |  | 3,766 (5.8%) | 617 (5.5%) |  |
| 65–69 | 8,236 (6.5%) | 1,333 (6.2%) |  | 3,999 (6.5%) | 638 (6.2%) |  | 4,237 (6.5%) | 696 (6.2%) |  |
| 70–74 | 9,189 (7.3%) | 1,614 (7.5%) |  | 4,337 (7.1%) | 750 (7.3%) |  | 4,852 (7.5%) | 865 (7.7%) |  |
| 75–79 | 7,065 (5.6%) | 1,286 (6.0%) |  | 3,146 (5.1%) | 565 (5.5%) |  | 3,918 (6.0%) | 720 (6.4%) |  |
| 80–84 | 5,404 (4.3%) | 942 (4.4%) |  | 2,232 (3.6%) | 388 (3.8%) |  | 3,172 (4.9%) | 553 (4.9%) |  |
| ≥85 | 6,133 (4.9%) | 1,018 (4.7%) |  | 1,927 (3.1%) | 318 (3.1%) |  | 4,207 (6.5%) | 700 (6.2%) |  |

Note: Percentages represent the proportion of each age group within the respective total or study population. Chi-square test performed for age group for men, women and both sexes.
