# Supplementary material for: Impact of COVID-19 on new pharmacotherapy for insomnia: A matched cohort study using the national insurance claims database in Japan
Source: PLoS One. 2026 Jan 22;21(1):e0341416. doi: 10.1371/journal.pone.0341416 (PMC12826487; doi:10.1371/journal.pone.0341416)
Supplement: S2 Table — (DOCX) [file pone.0341416.s002.docx]

Supplementary Table 2. Average per capita medical expenses related to mental illness in targeted prefectures

| **Prefecture** | **Mental-health cost per resident (¥ × 10³)** | **Rank among 47 prefectures†** | **Percentile** |
| --- | --- | --- | --- |
| **Shiga** (lowest) | 26.65 | 1 / 47 | 2ᵗʰ pctl |
| **Kyoto** | 29.89 | 8 / 47 | 17ᵗʰ pctl |
| **Osaka** | 31.65 | 11 / 47 | 23ᵗʰ pctl |
| **Hyōgo** | 33.08 | 15 / 47 | 32ᵗʰ pctl |
| **Okayama** | 37.43 | 22 / 47 | 47ᵗʰ pctl |
| **Tottori** (≈median) | 40.75 | 27 / 47 | 57ᵗʰ pctl |
| **Hiroshima** | 45.99 | 32 / 47 | 68ᵗʰ pctl |
| **Saga** (highest) | 62.69 | 47 / 47 | 100ᵗʰ pctl |
| †Rank 1 = lowest cost (Shiga 26.65 k); Rank 47 = highest cost (Saga 62.69 k).Lower end represented: Kyoto and Osaka sit in the lowest quintile of mental-health spending, comparable to Tokyo and Shiga. The national median (¥ 41k, Tottori). Higher-spending context included: Hiroshima’s cost (¥ 46 k) places it in the top third, approaching high-outlay prefectures such as Ishikawa and Yamagata. | | | |
