# Supplementary material for: Impact of COVID-19 on new pharmacotherapy for insomnia: A matched cohort study using the national insurance claims database in Japan
Source: PLoS One. 2026 Jan 22;21(1):e0341416. doi: 10.1371/journal.pone.0341416 (PMC12826487; doi:10.1371/journal.pone.0341416)
Supplement: S3 Table — (DOCX) [file pone.0341416.s003.docx]

Supplementary Table 3: ATC code, categories and drug names included in this study.

| **ATC** | **Category** | **Drugs** |
| --- | --- | --- |
| N05CD | **Short-acting Benzodiazepines (SA-BZO)** | Triazolam, Lormetazepam, Rilmazafone, Brotizolam |
|  | **Intermediate- and long- acting Benzodiazepines (ILA-BZO)** | Quazepam, Nitrazepam, Estazolam, Flunitrazepam, Flurazepam |
| N05CF | **Non-Benzodiazepine Hypnotics (Non-BZO)** | Eszopiclone, Zopiclone, Zolpidem |
| N05CH | **Melatonin Receptor Agonists (MRA)** | Ramelteon, Melatonin |
| N05CJ | **Orexin Receptor Antagonists (ORA)** | Lemborexant, Suvorexant |
| N05CA | **Barbiturates** | Pentobarbital |

ATC: anatomical therapeutic chemical.
