# Supplementary material for: Impact of COVID-19 on new pharmacotherapy for insomnia: A matched cohort study using the national insurance claims database in Japan
Source: PLoS One. 2026 Jan 22;21(1):e0341416. doi: 10.1371/journal.pone.0341416 (PMC12826487; doi:10.1371/journal.pone.0341416)
Supplement: S4 Table — (DOCX) [file pone.0341416.s004.docx]

| Supplementary Table 4: Incidence Rate Ratios and Differences of Pharmacotherapy for Insomnia after the COVID-19 infection in the Matched Cohort Design for Composite endpoint and subgroup categories. | | | | | |
| --- | --- | --- | --- | --- | --- |
|  | **No. of pair** | **No. of Events in COVID-19 infected Group** | **No. of Events in Control Group** | **Cumulative Incidence  (No. of Events per 1 000 000 Person months)** | |
|  |  |  |  | ***Ratio (95% CI)*** | ***Difference (95% CI)*** |
| Composite endpoint | 2226589 | 77626 | 43142 | 1.71 (1.69 – 1.73) | 1634 (1599 to 1669) |
| Subgroup |  |  |  |  |  |
| Sex |  |  |  |  |  |
| Men | 1004606 | 33746 | 17825 | 1.80 (1.77 – 1.84) | 1681 (1631 to 1732) |
| Women | 1221983 | 43880 | 25317 | 1.64 (1.62 – 1.67) | 1594 (1545 to 1642) |
| Age category |  |  |  |  |  |
| 0––19 | 485210 | 4540 | 2724 | 1.46 (1.39 – 1.53) | 380 (333 to 427) |
| 20––64 | 1009738 | 34009 | 21426 | 1.47 (1.45 – 1.50) | 1194 (1142 to 1246) |
| 65 or above | 731641 | 39077 | 18992 | 2.11 (2.07 – 2.14) | 2995 (2926 to 3063) |

No.: number; CI: confidence interval.
