# Supplementary material for: Impact of COVID-19 on new pharmacotherapy for insomnia: A matched cohort study using the national insurance claims database in Japan
Source: PLoS One. 2026 Jan 22;21(1):e0341416. doi: 10.1371/journal.pone.0341416 (PMC12826487; doi:10.1371/journal.pone.0341416)
Supplement: S5 Table — (DOCX) [file pone.0341416.s005.docx]

|  | Supplementary Table 5: Sensitivity Analysis of Pharmacotherapy for Insomnia after the COVID-19 infection in the Matched Cohort Design for Composite endpoint and subgroup categories. | | | | | | |
| --- | --- | --- | --- | --- | --- | --- | --- |
|  | | **Segmented interval** | **No. of individuals** | **No. of Events in COVID–19 Group** | **No. of Events in Control Group** | **Cumulative Incidence  (No. of Events per 1 000 000 Person months)** | |
|  |  |  |  |  |  | ***Ratio (95% CI)*** | ***Difference (95% CI)*** |
| Overall | | < 5 months | 4453178 | 35342 | 15168 | 2.33 (2.28 – 2.37) | 2581 (2525 to 2638) |
|  |  | 5–12 months | 3357507 | 28994 | 18761 | 1.46 (1.43 – 1.48) | 1077 (1025 to 1129) |
|  |  | 13 months or later | 1095671 | 13290 | 9213 | 1.25 (1.21 – 1.28) | 752 (662 to 842) |
| Subgroup | |  |  |  |  |  |  |
| Sex | |  |  |  |  |  |  |
| Male | | < 5 months | 2009212 | 15386 | 6180 | 2.49 (2.42 – 2.56) | 2615 (2533 to 2697) |
|  |  | 5–12 months | 1588774 | 12550 | 7687 | 1.54 (1.50 – 1.59) | 1151 (1076 to 1225) |
|  |  | 13 months or later | 500438 | 5810 | 3958 | 1.28 (1.23 – 1.33) | 791 (661 to 920) |
| Female | | < 5 months | 2443966 | 19956 | 8988 | 2.21 (2.16 – 2.27) | 2553 (2475 to 2631) |
|  |  | 5–12 months | 1848733 | 16444 | 11074 | 1.40 (1.36 – 1.43) | 1015 (942 to 1087) |
|  |  | 13 months or later | 595233 | 7480 | 5255 | 1.22 (1.18 – 1.27) | 717 (592 to 842) |
| Age category | |  |  |  |  |  |  |
| Age, 0–19 | | < 5 months | 970420 | 1826 | 1019 | 1.73 (1.60 – 1.87) | 449 (388 to 511) |
|  |  | 5–12 months | 827755 | 1938 | 1253 | 1.29 (1.20 – 1.38) | 266 (192 to 341) |
|  |  | 13 months or later | 142665 | 776 | 452 | 1.17 (1.04 – 1.31) | 260 (68 to 452) |
| Age, 20–64 | | < 5 months | 2019476 | 14389 | 7758 | 1.85 (1.80 – 1.90) | 1867 (1784 to 1949) |
|  |  | 5–12 months | 1521067 | 13260 | 9401 | 1.30 (1.26 – 1.33) | 771 (693 to 848) |
|  |  | 13 months or later | 498409 | 6360 | 4267 | 1.18 (1.13 – 1.22) | 588 (450 to 725) |
| Age, 65 or above | | < 5 months | 1463282 | 19127 | 6391 | 3.05 (2.97 – 3.14) | 5051 (4929 to 5174) |
|  |  | 5–12 months | 1008685 | 13796 | 8107 | 1.76 (1.72 – 1.81) | 2076 (1976 to 2175) |
|  |  | 13 months or later | 454597 | 6154 | 4494 | 1.37 (1.32 – 1.43) | 1171 (1030 to 1313) |

No.: number; CI: confidence interval
