# Supplementary material for: Impact of COVID-19 on new pharmacotherapy for insomnia: A matched cohort study using the national insurance claims database in Japan
Source: PLoS One. 2026 Jan 22;21(1):e0341416. doi: 10.1371/journal.pone.0341416 (PMC12826487; doi:10.1371/journal.pone.0341416)

**Supplementary Figure 1: Illustration of this study design**

In the matched cohort design, each COVID-19 infected individuals is matched on Charlson comorbidity index (CCI), and the month of enrollment to non-infected control individuals. Follow-up starts at index month, the month of infected COVID-19 and the matched month for the control. Individuals are followed until the outcome of interest, or the end of the observation period.


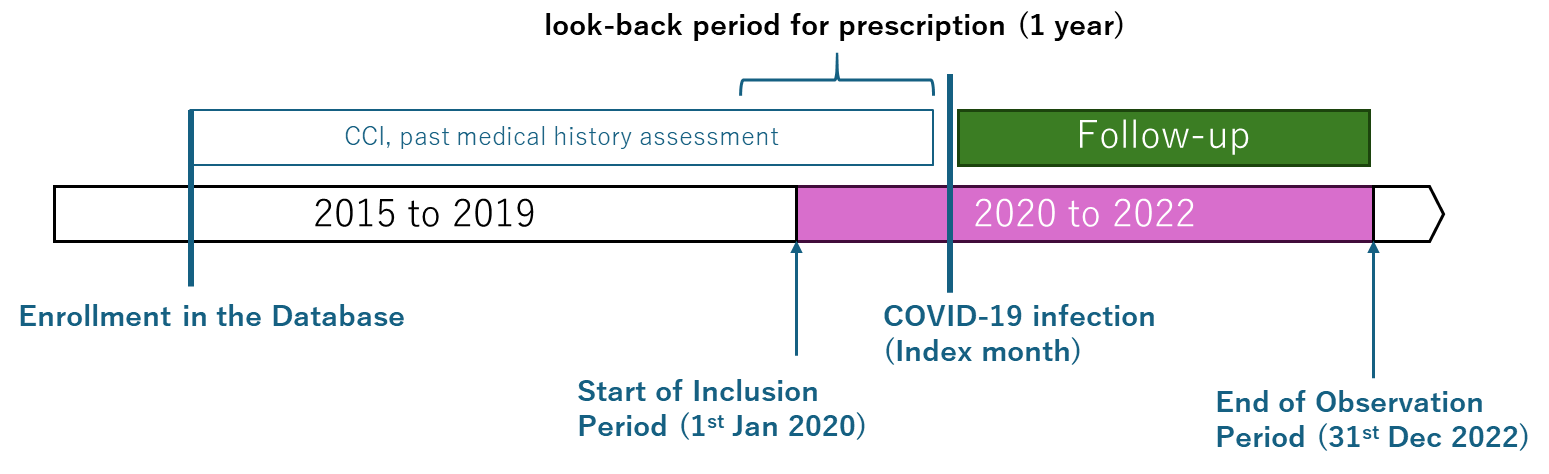

Supplement: S1 Fig — (DOCX) [file pone.0341416.s006.docx]
