# Supplementary material for: Impact of COVID-19 on new pharmacotherapy for insomnia: A matched cohort study using the national insurance claims database in Japan
Source: PLoS One. 2026 Jan 22;21(1):e0341416. doi: 10.1371/journal.pone.0341416 (PMC12826487; doi:10.1371/journal.pone.0341416)
Supplement: Attachment — Submitted filename: Response to reviewers.docx [file pone.0341416.s008.docx]

PONE-D-25-01134

Impact of COVID-19 on New Pharmacotherapy for Insomnia: A Matched Cohort Study using the National Insurance Claims Database in Japan

PLOS ONE

Dear Dr. Miyamori,

Thank you for submitting your manuscript to PLOS ONE. After careful consideration, we feel that it has merit but does not fully meet PLOS ONE’s publication criteria as it currently stands. Therefore, we invite you to submit a revised version of the manuscript that addresses the points raised during the review process.

Please submit your revised manuscript by May 03 2025 11:59PM. If you will need more time than this to complete your revisions, please reply to this message or contact the journal office at plosone@plos.org. When you're ready to submit your revision, log on to https://www.editorialmanager.com/pone/ and select the 'Submissions Needing Revision' folder to locate your manuscript file.

Please include the following items when submitting your revised manuscript:

A rebuttal letter that responds to each point raised by the academic editor and reviewer(s). You should upload this letter as a separate file labeled 'Response to Reviewers'.

A marked-up copy of your manuscript that highlights changes made to the original version. You should upload this as a separate file labeled 'Revised Manuscript with Track Changes'.

An unmarked version of your revised paper without tracked changes. You should upload this as a separate file labeled 'Manuscript'.

If you would like to make changes to your financial disclosure, please include your updated statement in your cover letter. Guidelines for resubmitting your figure files are available below the reviewer comments at the end of this letter.

If applicable, we recommend that you deposit your laboratory protocols in protocols.io to enhance the reproducibility of your results. Protocols.io assigns your protocol its own identifier (DOI) so that it can be cited independently in the future. For instructions see: https://journals.plos.org/plosone/s/submission-guidelines#loc-laboratory-protocols. Additionally, PLOS ONE offers an option for publishing peer-reviewed Lab Protocol articles, which describe protocols hosted on protocols.io. Read more information on sharing protocols at https://plos.org/protocols?utm_medium=editorial-email&utm_source=authorletters&utm_campaign=protocols.

We look forward to receiving your revised manuscript.

Kind regards,

Tatsuya Noda

Academic Editor

PLOS ONE

Journal Requirements:

When submitting your revision, we need you to address these additional requirements.

1. Please ensure that your manuscript meets PLOS ONE's style requirements, including those for file naming. The PLOS ONE style templates can be found at

https://journals.plos.org/plosone/s/file?id=wjVg/PLOSOne_formatting_sample_main_body.pdf and

<https://journals.plos.org/plosone/s/file?id=ba62/PLOSOne_formatting_sample_title_authors_affiliations.pdf>

Thank you for your suggestions regarding the requirements for PLOS ONE. We have formatted the manuscript as you suggested.

2. Thank you for stating the following financial disclosure: This study was financially supported by the Innovative Research Program on Suicide Countermeasures (Grant Number JPSCIRS20220305) and JSPS KAKENHI Grant-in-Aid for Early-Career Scientists (Number 21K17227).

Please state what role the funders took in the study. If the funders had no role, please state: ""The funders had no role in study design, data collection and analysis, decision to publish, or preparation of the manuscript.""

If this statement is not correct you must amend it as needed.

Please include this amended Role of Funder statement in your cover letter; we will change the online submission form on your behalf.

Thank you for your suggestions regarding the funder roles on the study. We have added the sentence as follows.

Funding, 2^nd^ sentence.

The funders had no role in study design, data collection and analysis, decision to publish, or preparation of the manuscript.

3. We note that you have indicated that there are restrictions to data sharing for this study. For studies involving human research participant data or other sensitive data, we encourage authors to share de-identified or anonymized data. However, when data cannot be publicly shared for ethical reasons, we allow authors to make their data sets available upon request. For information on unacceptable data access restrictions, please see http://journals.plos.org/plosone/s/data-availability#loc-unacceptable-data-access-restrictions.

Before we proceed with your manuscript, please address the following prompts:

a) If there are ethical or legal restrictions on sharing a de-identified data set, please explain them in detail (e.g., data contain potentially identifying or sensitive patient information, data are owned by a third-party organization, etc.) and who has imposed them (e.g., a Research Ethics Committee or Institutional Review Board, etc.). Please also provide contact information for a data access committee, ethics committee, or other institutional body to which data requests may be sent.

b) If there are no restrictions, please upload the minimal anonymized data set necessary to replicate your study findings to a stable, public repository and provide us with the relevant URLs, DOIs, or accession numbers. Please see http://www.bmj.com/content/340/bmj.c181.long for guidelines on how to de-identify and prepare clinical data for publication. For a list of recommended repositories, please see https://journals.plos.org/plosone/s/recommended-repositories. You also have the option of uploading the data as Supporting Information files, but we would recommend depositing data directly to a data repository if possible.

Please update your Data Availability statement in the submission form accordingly.

Thank you for your suggestions regarding the data availability statement. The data are owned by Ministry of Health, Lavour and Welfare in Japan and needs permission to use. Therefore, we have added the sentence as follows.

Ethical Consideration and Data Availability section, 2^nd^ sentence.

Data are owned by Ministry of Health, Labor, and Welfare in Japan (MHLW).

4. Please include captions for your Supporting Information files at the end of your manuscript, and update any in-text citations to match accordingly. Please see our Supporting Information guidelines for more information: http://journals.plos.org/plosone/s/supporting-information.

Thank you for your suggestions regarding the Supplemental data. We have added the captions for my supporting information at the end of the manuscript as follows.

Supporting Information

Supplementary Table 1. ATC code, categories and drug names included in this study.

Supplementary Table 2. Cumulative Incidences of Pharmacotherapy for Insomnia after the COVID-19 infection in the Matched Cohort Design for Composite endpoint, secondary endpoint and subgroup categories.

Supplementary Table 3. Cumulative Incidences of Pharmacotherapy for Insomnia after the COVID-19 infection in the Matched Cohort Design for Composite endpoint, secondary endpoint and subgroup categories.

Supplementary Figure 1. Illustration of this study design

Additional Editor Comments:

Thank you for your submission.

I believe that the reviewers' comments are all important points.

The authors should adequately respond to all of the reviewers' comments before reaching a decision on publication.

As an editor, I consider it necessary to revise some of the analyses.

I hope that an improved manuscript will be resubmitted.

Review Comments to the Author

Please use the space provided to explain your answers to the questions above. You may also include additional comments for the author, including concerns about dual publication, research ethics, or publication ethics. (Please upload your review as an attachment if it exceeds 20,000 characters)

Reviewer #1: The authors of this manuscript investigated the incidence of initiating pharmacological treatments for insomnia after COVID-19. I have several comments for this manuscript.

1. The authors need to cite appropriate previous studies and explain the scientific rationale on incidence of insomnia increase versus post-COVID-19 infection is so interesting. And then, authors need to support their study approach indicating the incidence of insomnia medications increase in individuals with post-COVID-19 infection can be reliable to measure that research question. However, the authors fail to explain the impact toward public health on COVID-19 infection and mental health which may be potentially a huge issue. These related statements remain fragile.

Thank you for your suggestions, as you indicated, we have changed the sentence as follows.

Page 5, Line80-100, Introduction, 2^nd^ paragraph

The scientific rationale for examining insomnia incidence post-COVID-19 infection stems from several factors. First, the neuroinvasive potential of SARS-CoV-2 may directly affect brain regions involved in sleep regulation [5]. Second, the psychological stress, social isolation, and lifestyle changes associated with the pandemic can disrupt sleep patterns [6]. Third, persistent symptoms of long COVID, such as fatigue and breathing difficulties, may interfere with sleep quality [7]. Additionally, investigating insomnia medication use provides an objective measure of clinically significant sleep disturbances requiring pharmacological intervention.

2. NDB is a medical claim database. Claimed disease name in these medical insurance databases is often different from diagnosed disease. Seems related validation study is not available and/or prepared for this study topic. The authors need to be cautious on how the data became generated. The authors need to explain why this study approach can reasonably identify the study patients with insomnia and COVID-19 infection.

Thank you for your suggestions regarding the potential bias due to the insurance claimed database.

We have added the sentence in the limitation section.

Page 23, Line 462-465, Strength and Limitations section, 2^nd^ paragraph, 4 and 5^th^ sentences.

Fourth, the codes identified in this study have not undergone validation. In research that assesses the accuracy of Japanese diagnostic codes for hospitalized patients, sensitivity and specificity were 78.9% and 93.2%, respectively [37].

3. Study patients are only from 5 prefectures; however, using NDB dataset. Please provide information related to methodologies that other researchers can reproduce this result. Furthermore, are these populations representable of nationwide Japan? Why did you choose these 5 prefectures instead of 47? I think regions with higher incidence rate and prevalence of mental health diseases are missing from this study population.

Thank you for your suggestions regarding the selection bias due to regional issues. As you indicated, we have change the sentence as follows:

Page 23, Line 471-475, Discussion, Strength and Limitation, 8^th^ sentence

Sixth, this study sampled only patients in the five prefectures, not in Japan as a whole. Although the population composition does not differ from that of Japan as a whole, the possibility cannot be denied that the risk of COVID-affected patients and the frequency of insomnia patients may differ from those in other prefectures (Supplementary Table4).

Supplementary Table 4. Populations in this study and Japan by Age Group and Sex

| **Age Group (years)** | **Population in Japan (N=126,146), n (%)** | **Study Population (N=21,569), n (%)** | **p-value** | **Men: Japan (N=61,350), n (%)** | **Men: Study Population (N=10,332), n (%)** | **p-value** | **Women: Japan (N=64,797), n (%)** | **Women: Study Population (N=11,237), n (%)** | **p-value** |
| --- | --- | --- | --- | --- | --- | --- | --- | --- | --- |
| 0–4 | 4,541 (3.6%) | 782 (3.6%) | 0.19 | 2,325 (3.8%) | 400 (3.9%) | 0.83 | 2,217 (3.4%) | 381 (3.4%) | 0.19 |
| 5–9 | 5,114 (4.1%) | 875 (4.1%) |  | 2,620 (4.3%) | 447 (4.3%) |  | 2,494 (3.8%) | 429 (3.8%) |  |
| 10–14 | 5,376 (4.3%) | 924 (4.3%) |  | 2,756 (4.5%) | 474 (4.6%) |  | 2,620 (4.0%) | 450 (4.0%) |  |
| 15–19 | 5,706 (4.5%) | 1,006 (4.7%) |  | 2,928 (4.8%) | 513 (5.0%) |  | 2,779 (4.3%) | 493 (4.4%) |  |
| 20–24 | 6,320 (5.0%) | 1,145 (5.3%) |  | 3,234 (5.3%) | 574 (5.6%) |  | 3,086 (4.8%) | 571 (5.1%) |  |
| 25–29 | 6,384 (5.1%) | 1,093 (5.1%) |  | 3,279 (5.3%) | 547 (5.3%) |  | 3,105 (4.8%) | 546 (4.9%) |  |
| 30–34 | 6,714 (5.3%) | 1,124 (5.2%) |  | 3,431 (5.6%) | 564 (5.5%) |  | 3,283 (5.1%) | 561 (5.0%) |  |
| 35–39 | 7,498 (5.9%) | 1,232 (5.7%) |  | 3,806 (6.2%) | 611 (5.9%) |  | 3,692 (5.7%) | 621 (5.5%) |  |
| 40–44 | 8,476 (6.7%) | 1,411 (6.5%) |  | 4,299 (7.0%) | 697 (6.7%) |  | 4,178 (6.4%) | 714 (6.4%) |  |
| 45–49 | 9,868 (7.8%) | 1,728 (8.0%) |  | 4,994 (8.1%) | 854 (8.3%) |  | 4,875 (7.5%) | 872 (7.8%) |  |
| 50–54 | 8,738 (6.9%) | 1,516 (7.0%) |  | 4,394 (7.2%) | 747 (7.2%) |  | 4,344 (6.7%) | 770 (6.9%) |  |
| 55–59 | 7,940 (6.3%) | 1,336 (6.2%) |  | 3,967 (6.5%) | 655 (6.3%) |  | 3,973 (6.1%) | 683 (6.1%) |  |
| 60–64 | 7,442 (5.9%) | 1,200 (5.6%) |  | 3,677 (6.0%) | 584 (5.7%) |  | 3,766 (5.8%) | 617 (5.5%) |  |
| 65–69 | 8,236 (6.5%) | 1,333 (6.2%) |  | 3,999 (6.5%) | 638 (6.2%) |  | 4,237 (6.5%) | 696 (6.2%) |  |
| 70–74 | 9,189 (7.3%) | 1,614 (7.5%) |  | 4,337 (7.1%) | 750 (7.3%) |  | 4,852 (7.5%) | 865 (7.7%) |  |
| 75–79 | 7,065 (5.6%) | 1,286 (6.0%) |  | 3,146 (5.1%) | 565 (5.5%) |  | 3,918 (6.0%) | 720 (6.4%) |  |
| 80–84 | 5,404 (4.3%) | 942 (4.4%) |  | 2,232 (3.6%) | 388 (3.8%) |  | 3,172 (4.9%) | 553 (4.9%) |  |
| ≥85 | 6,133 (4.9%) | 1,018 (4.7%) |  | 1,927 (3.1%) | 318 (3.1%) |  | 4,207 (6.5%) | 700 (6.2%) |  |

Note: Percentages represent the proportion of each age group within the respective total or study population. Chi-square test performed for age group for men, women and both sexes.

4. Table 1: Was the total study patients (N=4.4 million) reasonable level for explaining the incidence/ prevalence of insomnia among these 5 prefectures?

Thank you for your suggestions, regarding the study populations in this study. Since matching process might reduced the generalizability in this study, we have added the sentence in the limitation section.

Page 23, Line 468-471, Discussion, Strength and Limitations section, 2^nd^ paragraph, 6^th^ sentence.

Fifth, despite efforts to reduce confounding through matching on key variables, the study may still be subject to residual confounding from unmeasured factors not accounted for in the matching process, potentially biasing the results and limiting generalizability to broader populations.

5. The authors need to provide scientifically reasonable information on why the outcomes are defined appropriately in this study settings since claimed disease and actual diagnosed disease are often different.
6. The authors need to provide scientifically reasonable information on why the prescriptions were made only for insomnia in this study settings and made it possible to exclude other indications.

Thank you for pointing this out. In defining medication in this study, benzodiazepines and antidepressants indicated for anxiety and other conditions were not used as outcomes. We have added a description of this in the Outcome section. The results in the text have changed slightly because the matching was performed again accordingly.

Page 8, Line 159-167, Outcome section,1^st^ and 2^nd^ sentences.

The outcome of interest of this study was the initiation of prescription for insomnia which are available for outpatients in Japan, with insomnia being the main indication, and other indications such as anxiety and epilepsy being excluded. We categorize the drug as follows: Melatonin Receptor Agonist (MRA), Non-Benzodiazepine Hypnotic (non-BZO), benzodiazepine (short-acting [SA-BZO], intermediate-/long-acting [ILA-BZO]), Orexin Receptor Antagonist (ORA) and Barbiturates.

Supplementary Table 1: ATC code, categories and drug names included in this study.

| **ATC** | **Category** | **Drugs** |
| --- | --- | --- |
| N05CD | Short-acting Benzodiazepines (SA-BZO) | Triazolam, Lormetazepam, Rilmazafone, Brotizolam |
|  | Intermediate- and long- acting Benzodiazepines (ILA-BZO) | Quazepam, Nitrazepam, Estazolam, Flunitrazepam, Flurazepam |
| N05CF | Non-Benzodiazepine Hypnotics (Non-BZO) | Eszopiclone, Zopiclone, Zolpidem |
| N05CH | Melatonin Receptor Agonists (MRA) | Ramelteon, Melatonin |
| N05CJ | Orexin Receptor Antagonists (ORA) | Lemborexant, Suvorexant |
| N05CA | Barbiturates | Pentobarbital |

ATC: anatomical therapeutic chemical.

7. The authors calculated CCI scores based on claimed insurance disease which is different from actual diagnosed disease. Please cite appropriate previous studies and explain how this approach is scientifically reasonable and reliable.

Thank you for your suggestions regarding CCI scores on this study, as you indicated we have added the sentence in the limitation section.

Page 24, Line 469-472, Strength and Limitations section, 2^nd^ paragraph, 6^th^ sentences.

Although the validity of each disease was not determined, the CCI score calculated using the Japanese health insurance database was reported to be around 0.7-0.85 for c-statistics, which we believe indicates a certain validity of the background diseases in this study [38, 39].

Reviewer #2: Thank you for the opportunity to review the manuscript. I believe that several points need to be addressed.

Major issues:

1. The authors should address the potential for differential misclassification bias related to insomnia. It is plausible that the majority of patients do not receive treatment for insomnia, and individuals in the exposure group may be more likely to receive such treatment compared to those in the control group, potentially due to differences in help-seeking behavior.

I have added this to the Discussion section, as the impact as help seeking behavior you pointed out is indeed relevant.

Page 19, Line 370-376, Discussion section, 4^th^ paragraph,

Furthermore, individuals with COVID-19 might be at an elevated risk of seeking medical attention compared to those without the disease, due to the diverse range of symptoms they exhibit [27]. Moreover, the public health emphasis on early detection and isolation of COVID-19 cases may contribute to this increased healthcare-seeking behavior [28]. Individuals experiencing symptoms consistent with COVID-19 may be more likely to reach out to healthcare providers for testing, diagnosis, and guidance on appropriate management and isolation measures [29].

2. The authors concluded that the initiation of insomnia medication highlights the necessity of mental health support. However, clinical guidelines for the treatment of insomnia generally recommend nonpharmacological interventions as the first-line approach. Therefore, the initiation of pharmacological treatment for insomnia does not necessarily indicate a need for mental health support, but may instead reflect potentially inappropriate prescribing practices.

In Japan, cognitive-behavioral therapy is not the primary treatment option, as it is in other countries, and is only recommended for certain patients according to the guidelines. This issue may be linked to the study's findings, which align with the projected increase in insomnia cases, and could be associated with the elevated risk of benzodiazepine dependence in Japan. We have incorporated this point into the Discussion section.

Page 17, Line 322-328, Discussion section, 2^nd^ paragraph, 4-6^th^ sentences.

In addition, in Japan, cognitive-behavioral therapy for sleep disorder is not covered by insurance and is not reimbursed. In addition, the 2014 Japanese Practical Guidelines for Insomnia do not recommend cognitive-behavioral therapy as a first line therapy, and consider treatment when there is little improvement with pharmacotherapy[22]. In fact, these are the reasons why cognitive-behavioral therapy has not been widely used in Japan, e.g., reports indicate that only 60,000 patients nationwide received CBT for mood disorders between 2010 and 2015 [23].

Page 21, Line 416-420, Discussion, 5^th^ paragraph, 1^st^ and 2^nd^ last sentences.

Conversely, while cognitive behavioral therapy is the preferred treatment internationally, the rising use of sleeping pills in Japan may indicate a current demand for psychological support. However, it may also suggest an increase in the incidence of inappropriate prescribing, as previously mentioned.

3. The authors stated that the rationale for this study was to investigate the long-term effects of COVID-19 on mental health. However, the median follow-up period was only seven months. The authors should clarify the justification for this follow-up duration in the context of evaluating long-term outcomes.

Thank you. As you mentioned, the median time in this study was 7 months, which may not be considered a long period of time. Therefore, I have removed the ”long-term” part from the text.

4. The authors identified insomnia medications using ATC codes. For transparency and reproducibility, please provide a complete list of the included drug names, their routes of administration, and corresponding domestic drug codes. In particular, clarification is needed on the following points: What is 'lilmazepam'?; Why were 'rilmazafone' and 'melatonin' not included?; How was 'haloxazolam' identified, given that it does not have an ATC code?; How were 'midazolam' and 'haloxazolam' excluded from the ATC category NC05CD?; Was flunitrazepam injection included in the analysis?; Why were other insomnia medications, such as barbiturates, not considered?

Thank you for your observations. As you noted, we have rectified certain errors. Furthermore, we have not utilized injectable drugs at this stage and have included an additional note in the methodology section to reflect this.

Page 8, Line 159-167, Methods, Outcome section, 1st and 2nd sentences.

Outcome: The outcome of interest of this study was the initiation of prescription for insomnia which are available for outpatients in Japan, with insomnia being the main indication, and other indications such as anxiety and epilepsy being excluded. We categorize the drug as follows: Melatonin Receptor Agonist (MRA), Non-Benzodiazepine Hypnotic (non-BZO), benzodiazepine (short-acting [SA-BZO], intermediate-/long-acting [ILA-BZO]), Orexin Receptor Antagonist (ORA) and Barbiturates.

Supplementary Table1

Supplementary Table 1: ATC code, categories and drug names included in this study.

| **ATC** | **Category** | **Drugs** |
| --- | --- | --- |
| N05CD | **Short-acting Benzodiazepines (SA-BZO)** | Triazolam, Lormetazepam, Rilmazafone, Brotizolam |
|  | **Intermediate- and long- acting Benzodiazepines (ILA-BZO)** | Quazepam, Nitrazepam, Estazolam, Flunitrazepam, Flurazepam |
| N05CF | **Non-Benzodiazepine Hypnotics (Non-BZO)** | Eszopiclone, Zopiclone, Zolpidem |
| N05CH | **Melatonin Receptor Agonists (MRA)** | Ramelteon, Melatonin |
| N05CJ | **Orexin Receptor Antagonists (ORA)** | Lemborexant, Suvorexant |
| N05CA | **Barbiturates** | Pentobarbital |

ATC: anatomical therapeutic chemical.

6. PLOS authors have the option to publish the peer review history of their article (what does this mean?). If published, this will include your full peer review and any attached files.

If you choose “no”, your identity will remain anonymous but your review may still be made public.

Do you want your identity to be public for this peer review? For information about this choice, including consent withdrawal, please see our Privacy Policy.

Reviewer #1: No

Reviewer #2: No

[NOTE: If reviewer comments were submitted as an attachment file, they will be attached to this email and accessible via the submission site. Please log into your account, locate the manuscript record, and check for the action link "View Attachments". If this link does not appear, there are no attachment files.]

While revising your submission, please upload your figure files to the Preflight Analysis and Conversion Engine (PACE) digital diagnostic tool, https://pacev2.apexcovantage.com/. PACE helps ensure that figures meet PLOS requirements. To use PACE, you must first register as a user. Registration is free. Then, login and navigate to the UPLOAD tab, where you will find detailed instructions on how to use the tool. If you encounter any issues or have any questions when using PACE, please email PLOS at figures@plos.org. Please note that Supporting Information files do not need this step.

In compliance with data protection regulations, you may request that we remove your personal registration details at any time. (Remove my information/details). Please contact the publication office if you have any questions.
