# Supplementary material for: Impact of COVID-19 on new pharmacotherapy for insomnia: A matched cohort study using the national insurance claims database in Japan
Source: PLoS One. 2026 Jan 22;21(1):e0341416. doi: 10.1371/journal.pone.0341416 (PMC12826487; doi:10.1371/journal.pone.0341416)
Supplement: Attachment — Submitted filename: Response to reviewers_R2.docx [file pone.0341416.s009.docx]

*6. Review Comments to the Author

Please use the space provided to explain your answers to the questions above. You may also include additional comments for the author, including concerns about dual publication, research ethics, or publication ethics. (Please upload your review as an attachment if it exceeds 20,000 characters)*

*Reviewer #1: The authors responded to the raised comments though still not sufficient to improve the scientific sound.*

*1. The authors sticks on showing CCI scores which is NOT reliable enough. The authors failed to explain and justify how the results related to CCI can be implemented in clinical practice regarding their research question. For instance, CCI is often used for considering the conditions of frailty in clinical practice. It is unclear why the authors presented the results in this study on CCI which is NOT reliable enough. How does this result contribute to clinical practice? Its implementation remains totally vague.*

**Response:** Thank you for your suggestions regarding the clinical implication of CCI in the clinical practice. We have added the sentences as you indicated.

Page 19, Line 303-307, Discussion section 5^th^ paragraph, 1-3 sentences.

Critically, the NDB does not contain the functional, cognitive, or social variables required to construct formal frailty indices, nor the complete problem lists needed for standard multimorbidity counts. Consequently, we used CCI as a pragmatic surrogate, since higher CCI scores correlate well with frailty and multimorbidity [34, 35]. As in previous studies, there was an increase in both younger and older age groups.

Page 19-20, Line 315-321, Discussion section 5^th^ paragraph, last 2 sentences.

These multimorbid, functionally vulnerable individuals account for the largest excess burden of post-COVID insomnia pharmacotherapy and are also most susceptible to hypnotic-related harms (falls, delirium, functional decline). Embedding brief sleep screening, fall-prevention counselling, and medication reviews into routine visits for this subgroup provides a pragmatic, resource-efficient way to translate our findings into clinical care, even where real-time CCI calculation is not feasible.

*2. Introduction is too long. Some statement is NOT directly related to the research question. The authors need to focus on appropriate citation to support their storyline with appropriate length.*

Thank you for your suggestions, as you indicated we have deleted redundant sentences and simplify the statement for aims in the Introduction section.

| Prefecture | Mental-health cost per resident (¥ × 10³) | Rank among 47 prefectures† | Percentile |
| --- | --- | --- | --- |
| Kyoto | 28.96 | 4 / 47 | 9ᵗʰ pctl (lowest decile) |
| Osaka | 29.73 | 7 / 47 | 15ᵗʰ pctl |
| Hyōgo | 33.45 | 13 / 47 | 28ᵗʰ pctl |
| Okayama | 40.48 | 21 / 47 | 45ᵗʰ pctl |
| Hiroshima | 47.34 | 33 / 47 | 70ᵗʰ pctl (upper tertile) |
| †Rank 1 = lowest cost (Tokyo 27.62 k); Rank 47 = highest cost (Saga 68.74 k).Lower end represented: Kyoto and Osaka sit in the lowest quintile of mental-health spending, comparable to Tokyo and Shiga. Median captured: Okayama’s expenditure (¥ 40 k) is almost identical to the national median (¥ 41 k, Fukushima). Higher-spending context included: Hiroshima’s cost (¥ 47 k) places it in the top third, approaching high-outlay prefectures such as Ishikawa and Yamagata. | | | |

*3. Generalizability remains doubtful. The authors need to add statements related to external validity. Is the result consistent with previous results? What will be the perspectives of the study group, related to the interpretation of the results? Why is the statement related to the interpretation reasonable? These discussion remains fragile.*

Thank you for your suggestions, regarding the generalizability in our study. As you indicated we have added discussion section for the comparison with previous studies, and perspectives with interpretation of our study results.

Page 8, Line 116-119, Methods, Study participants, 3^rd^ sentence.

In the five prefectures included in this study, the age-demographic composition was similar to that of the nation as a whole, and the cost frequency for psychiatric disorders as of 2021 did not show extreme bias. (Supplementary Tables 1 and 2).

Supplemental Table 2. Average per capita medical expenses related to mental illness in targeted prefectures

Page 9, Line 143-145, Methods, Data collection section, 2^nd^ sentence; Japan's universal health insurance system features a detailed NDB that thoroughly records each outpatient appointment, hospital stay, diagnostic code, and medication prescribed for all residents.

Page 16, Line 255-164, Discussion section, 2^nd^ paragraph

Previous studies have reported a sharp increase in hyponotics immediately after the COVID-19 epidemic, with an increase of 0.23 boxes per 1000 persons, [4] and a risk of being diagnosed with insomnia about 1.92 times higher six months after the illness and 2.46 times higher even one year later. [22] [23] It has also been reported that 30% of patients with severe PASC required sleep-inducing drugs for an extended period of time. [24]Using the Population database, which is a highly complete database, the present study observed a longer period of time than previous studies and found that the risk after COVID-19 disease persisted for more than 2 years.Hypnotics, medications generally used to treat insomnia, may be more frequently prescribed to manage persistent sleep-related symptoms in these patients.

Page 17,Line 272-275, Discussion section, 3^rd^ paragraph

Based on our findings, in the context of Japan's unique clinical approach, [26] it is assumed that our results reflect the number of new pharmacotherapies for insomnia. Conversely, it is important to consider that the variations in treatment interventions for sleeping pills in Japan may limit the generalizability of this study.

Page 17-18, Line 277-286, Discussion section, 4^th^ paragraph, 1-5^th^ sentences.

No previous studies have examined which sleeping medications are more likely to be used for insomnia after COVID-19. The secondary endpoint showed a high rate of increase in the non-BZO and MRA categories, while a certain number of BZOs were also prescribed..The amount of BZO and non-BZO prescriptions in existing patients in recent years is reported to be 60% and 37%, indicating that the frequency of BZO prescriptions is still high in Japan.[27] Historical Japanese prescription patterns for new sleep medications using NDB from 2010-2019 showed that BZOs accounted for 30-50% of prescriptions, ORAs for 20%, and MRAs for about 3-6% of prescriptions[28]。In the current study, ORAs accounted for about 40% of the total, and MRAs were also increasing in prescription volume, at 10%. Japan has a high prevalence of benzodiazepine abuse disorders[29].

Page 18, Line 290-293, Discussion section, 4^th^ paragraph, last two sentences.

The results of this study suggest that the frequency of BZO prescriptions may be decreasing compared to the past. However, the high incidence of benzodiazepine dependence in Japan should continue to be monitored, which necessitates future improvement.

Page 23, Line 378-382, Discussion section, last paragraph, 4^th^ and 5^th^ last sentences.

Sixth, this study sampled only patients in the five prefectures, not in Japan as a whole, and the possibility cannot be denied that the risk of COVID-affected patients and the frequency of insomnia patients may differ from those in other prefectures. Similarly, generalizability is limited in foreign countries with different cultural backgrounds and treatment policies.

*Reviewer #2: Thank you for the opportunity to review the manuscript. I believe that a minor issue need to be addressed.

Minor issues:
1. The authors should address the potential for differential misclassification bias related to insomnia. It is plausible that the majority of patients do not receive treatment for insomnia, and individuals in the exposure group may be more likely to receive such treatment compared to those in the control group, potentially due to differences in help-seeking behavior.

[Author’s response] I have added this to the Discussion section, as the impact as help seeking behavior you pointed out is indeed relevant.

Page 19, Line 370-376, Discussion section, 4th paragraph,
Furthermore, individuals with COVID-19 might be at an elevated risk of seeking medical attention compared to those without the disease, due to the diverse range of symptoms they exhibit [27]. Moreover, the public health emphasis on early detection and isolation of COVID-19 cases may contribute to this increased healthcare-seeking behavior [28]. Individuals experiencing symptoms consistent with COVID-19 may be more likely to reach out to healthcare providers for testing, diagnosis, and guidance on appropriate management and isolation measures [29].

[Additional comments] The authors did not address misclassification bias (i.e., the potential for bias to lead to distorted estimates), but rather discussed only potential sources of bias. Please clarify the likely direction and magnitude of the misclassification bias.*

Thank you for your suggestions regarding the potential misclassification in our study. We have added the sentence as follows.

Page 22, Discussion, 2nd last paragraph, 2nd and 4th sentences.

Second, selection bias may exist due to misclassification of COVID-19 infection status and the outcome status.

Conversely, insomnia may be more frequently prescribed as a health-seeking behavior in patients affected by post-COVID-19 conditions, potentially amplifying the observed results.
