# Supplementary material for: Impact of COVID-19 on new pharmacotherapy for insomnia: A matched cohort study using the national insurance claims database in Japan
Source: PLoS One. 2026 Jan 22;21(1):e0341416. doi: 10.1371/journal.pone.0341416 (PMC12826487; doi:10.1371/journal.pone.0341416)
Supplement: Attachment — Submitted filename: 251204Plosone_pointbypoint.docx [file pone.0341416.s011.docx]

Reviewer #1 Comment:

*The question "Why did you present CCI in this manuscript?" remains not answered yet…*

Response

We sincerely thank the reviewer for this critical and constructive comment, which prompted us to reconsider the role and interpretation of the Charlson Comorbidity Index (CCI) in our manuscript.

We fully agree that higher CCI scores and older age are well-established correlates of increased medication use in general, and that presenting CCI as an independent risk factor for insomnia initiation does not constitute a novel or clinically actionable finding in itself. In this respect, we acknowledge that our previous presentation did not sufficiently clarify the rationale for including CCI-related results, nor did it adequately align with the primary research question of this study.

The primary purpose of incorporating CCI in this study was methodological rather than inferential. Specifically, CCI was used as a matching variable to balance baseline comorbidity burden between the COVID-19 and non-COVID-19 cohorts, thereby reducing confounding and isolating the effect of COVID-19 infection on subsequent initiation of insomnia pharmacotherapy. This design choice was essential to ensure internal validity, particularly given the strong association between multimorbidity and healthcare utilization.

Upon careful reconsideration of the reviewer’s concern, we concluded that presenting CCI-based subgroup analyses and discussing CCI as a clinical risk factor could distract from the central and novel message of this study—namely, that COVID-19 infection is associated with a substantially increased risk of initiating insomnia pharmacotherapy across all age groups, including children, adolescents, and younger adults, independent of baseline comorbidity burden.

In addition, in response to the reviewer’s concern that age-related findings may be interpreted as a reiteration of common clinical knowledge, we have removed all discussions that framed older age itself as a clinically meaningful or noteworthy risk factor for the initiation of insomnia pharmacotherapy.

Accordingly, we have revised the manuscript as follows: We removed all statements interpreting higher CCI scores as an independent risk factor for insomnia medication initiation. Furthermore, we deleted statements suggesting that older individuals were at higher risk per se, as such interpretations do not align with the primary objective of this study and may obscure the novel contribution of our findings.

The following CCI and older individuals-related interpretations have been removed:

・Abstract: references to increased risk among older individuals (Page 4, Lines 53–54).

・Methods: subgroup analysis for CCI categories (Page 11, Lines 170-171)

・Results: descriptive or interpretative statements emphasizing higher incidence in older age groups (Page 13, Lines 201; Page 14, Line 221-222).

・Discussion: age-centered interpretations implying that insomnia risk after COVID-19 is predominantly driven by older age (Page 15, Lines 240–242 and 246; Page 17, Line 286).

CCI is now explicitly described only as a covariate used for matching and confounding control, without further clinical interpretation.

By doing so, we sharpened the focus of the manuscript on its key and clinically relevant finding: that post-COVID-19 insomnia requiring pharmacological treatment is not limited to traditionally “high-risk” populations (e.g., older or multimorbid individuals), is also evident in younger and otherwise healthier populations. This has important implications for clinical practice, as it supports the need for vigilance and proactive screening for sleep disturbances in all patients following COVID-19, rather than targeting only those with advanced age or multiple comorbidities.

We believe that this revision directly addresses the reviewer’s concern and substantially strengthens the coherence, novelty, and clinical relevance of the manuscript.

In addition, in response to the reviewer’s concern that age-related findings may be interpreted as a reiteration of common clinical knowledge, we have removed all discussions that framed older age itself as a clinically meaningful or noteworthy risk factor for the initiation of insomnia pharmacotherapy.
